# Patient Attitudes About Light Therapy and Negative Ion Therapy for Nonseasonal Depression: An Online Survey Study

**DOI:** 10.7759/cureus.98278

**Published:** 2025-12-01

**Authors:** Orli S Hellerstein, Iman Lahouaoula, Victor W Li, Aidan Scott, Andre Do, Erin E Michalak, Jill K Murphy, Samantha Huang, Vanessa K Evans, Raymond W Lam

**Affiliations:** 1 Psychiatry, University of British Columbia, Vancouver, CAN; 2 Psychiatry, University of British Columbia, Faculty of Medicine, Vancouver, CAN; 3 Psychiatry, BC Children's Hospital, Vancouver, CAN; 4 Psychiatry, Universite de Montreal, Montreal, CAN; 5 Health, St. Francis Xavier University, Antigonish, CAN

**Keywords:** feasibility, ion therapy, light therapy, maintenance treatment, major depressive disorder (mdd), relapse prevention, survey

## Abstract

Objective: No studies have evaluated the feasibility of light therapy or negative ion therapy as maintenance treatments after acute antidepressant treatment in major depressive disorder. To address this gap, we surveyed people with depression about their knowledge and attitudes about light therapy and negative ion therapy, and their willingness to participate in a clinical trial of maintenance treatment with these therapies.

Methods: Participants with a self-reported diagnosis of depression completed a researcher-generated online survey, created for this study, examining awareness and effectiveness of light therapy and negative ion therapy, which included vignettes describing the use of these therapies for maintenance treatment. Participants were asked about the feasibility and reasons for wanting (and not wanting) to use the therapies instead of antidepressants. Response frequencies were compared using chi-square tests.

Results: A total of 193 participants completed the survey. Most were aware of both therapies, but significantly more participants had heard of light therapy (95% versus 63% for negative ion therapy, p<0.001), had used light therapy (29% versus 17%, p<0.001), and regarded light therapy as effective (54% versus 37%, p<0.001). Both therapies were considered easy to use. Most participants (81%) placed importance on finding non-medication therapies for maintenance treatment; 77% responded that they would likely volunteer for a randomized study of maintenance treatment.

Conclusion: People with depression are aware of light therapy and negative ion therapy and support their usage as substitutes for antidepressants in maintenance treatment. This supports the importance and feasibility of a randomized relapse prevention trial with light therapy and negative ion therapy in people with depression.

## Introduction

Major depressive disorder (MDD) affects approximately 264 million people worldwide and is one of the top three medical causes of years lived with disability [1]. Amongst the many evidence-based treatment options for MDD, antidepressants are recommended as first-line treatments for moderate to severe depression [2]. Once patients have recovered from an acute depressive episode, maintenance antidepressant treatment is recommended for 6-24 months, or longer, to prevent relapse and recurrence [2]. Several meta-analyses of randomized controlled trials (RCTs) have confirmed that maintenance antidepressants are effective in preventing relapse [3-5].

Despite the evidence for maintenance, patients often discontinue antidepressants too early after acute treatment [6]. This puts them at risk of relapse and consequent impairment in functioning and quality of life. Some studies suggest that less than 30% of patients continue antidepressants for more than three months [7]. Some patients stop antidepressants prematurely due to persistent side effects, including sexual dysfunction and weight gain; cost; adverse effects with long-term use of antidepressants (e.g., osteoporosis, gastrointestinal bleeding, and risk of drug interactions with medications for other medical conditions); and patient preference for non-medication treatments [8].

Given the reluctance for longer-term use of antidepressants, perhaps patients would consider using non-medication treatments such as bright light therapy and negative ion therapy to substitute for antidepressants during maintenance treatment. Light therapy involves daily exposure to bright light (e.g., 10,000 lux) from a light device usually constructed with fluorescent bulbs or light-emitting diodes [9]. Light therapy has long been considered a first-line treatment for seasonal affective disorder (SAD) [10,11], and increasing evidence supports its efficacy in nonseasonal MDD [12-14] and bipolar depression [15,16]. Negative ion therapy is delivered with a desktop ion generator, similar to an air purifier, that produces invisible air ions at a flow rate of 4.5 x 1014 ions/second or higher [17]. Studies of high-density negative ion treatment have also shown positive results in reducing depressive symptoms [18-21]. Both these treatments are safe and well-tolerated, with mild side effects that are less frequent and less severe than with antidepressants [9,19,22].

While light therapy and negative ion therapy seem promising for antidepressant substitution, neither treatment has been studied for maintenance treatment or relapse prevention. Rigorous RCTs are needed to demonstrate efficacy and safety, but it is unclear whether patients would find light therapy and negative ion therapy acceptable substitutes for maintenance treatments, given that they are not widely used in clinical practice. Patient engagement is now an established principle in all aspects of health research, including formulating research questions [23]. The current study examined participants' self-reporting a diagnosis of depression to determine (1) the importance of alternative non-medication treatment of MDD from a patient perspective, (2) their familiarity with light and ion therapy, and (3) the feasibility of conducting an RCT with light and ion therapy for maintenance treatment of MDD. A previous version of this article was deposited at the MedRxiv preprint server [24].

## Materials and methods

Participants

This study received approval from the Behavioural Research Ethics Board at the University of British Columbia (H20-01244). This exploratory, cross-sectional survey study was conducted with a convenience sample between July 17th, 2020, and July 7th, 2021. Participants were recruited from various sources, including social media postings, newsletters, and research networks in Canada. Inclusion criteria were (1) age 19-65 years; (2) diagnosed with depression by a physician or psychologist, by self-report; (3) capable of informed consent; (4) access to an internet-enabled computer or mobile device; and (5) able to read and understand English. Any participants who did not meet the inclusion criteria were excluded.

Assessments

The authors created a questionnaire for this study, comprised of 3 sections (see Appendix). Section 1 included 16 questions on participant demographics, whether they had been diagnosed with depression by a physician or psychologist, and whether they were taking, or had taken in the past, antidepressant medications.

Section 2 contained four questions addressing self-reported knowledge, opinions, and attitudes about light and ion treatments. Information stems were first presented, such as “Light therapy is a treatment for depression that uses daily exposure to bright light from a light box device used at home. Light therapy usually has fewer side effects than antidepressant medications,” followed by the questions “Have you heard of light therapy for depression?” and “In your opinion, how effective is light therapy for depression treatment?” The latter question included the responses “More effective than antidepressants,” “As effective as antidepressants,” “Less effective than antidepressants,” “Not effective,” and “Unsure.” A similar stem and the same questions were presented for negative ion therapy.

Section 3 contained 11 questions addressing substitution treatments for antidepressants in maintenance treatment of depression (see Appendix). Following a vignette for antidepressant maintenance treatment, participants were asked, “In your opinion, how important is it to substitute an evidence-based non-medication treatment for antidepressants for maintenance treatment?” with responses of “Very important,” “Quite important,” “Somewhat important,” and “Not at all important.” Then, two vignettes with identical phrasing were presented for substituting light and ion therapy for maintenance treatment, each followed by a question, “How easy would it be for you to use light/ion therapy as described?”

The survey also included vignettes (Appendix, Vignettes 4 and 5) to illustrate an individual’s use of light/ion therapy, describing the cost, time frame, and schedule, each followed by a question, “How easy would it be for you to use light/ion therapy as described?” with responses on a 7-point Likert scale ranging from “Very easy” to “Very difficult.” Open-ended questions asked participants to write reasons why they would want or would not want the treatments. A final vignette was presented with the procedures for an RCT of active versus inactive treatment with light and ion therapy for maintenance treatment (Appendix, Vignette 6), followed by a question asking, “How likely would you be to volunteer for this study?” with responses on a 7-point Likert scale ranging from “Very likely” to “Very unlikely.”

Procedure

All survey data were collected electronically using Qualtrics. Participants provided informed consent before starting the online survey.

Statistical analysis

All statistical analysis was conducted using IBM Corp. Released 2020. IBM SPSS Statistics for Windows, Version 29.0. Armonk, NY: IBM Corp. [25]. As this was an exploratory study, there was no precalculated sample size. The survey data are reported in counts and percentages, not including missing data. For some statistical comparisons of frequency of responses between groups, response categories were combined to create a unified measure, e.g., for perception of effectiveness of the treatments, we deemed treatment as “effective” by combining responses of “as effective as antidepressants” and “more effective than antidepressants.” Chi-square (χ2) tests were used to compare frequencies, with a significance level p set at less than 0.05.

## Results

A total of 221 individuals responded to the survey. Of these individuals, 193 self-reported a diagnosis of depression and are included in the analysis. Table 1 shows the demographic data of this sample. The majority of the depressed sample identified as women (n=125, 65%), reported an age in the 25-49 years bracket (n=115, 60%), self-identified as white/European (n=141, 73%), had an undergraduate or postgraduate/professional degree (n=112, 58%), were employed (n=141 of 191 responses, 74%), and lived in an urban area (n=105 of 190 responses, 55%).

**Table 1:** Demographic information

Category	Responses	Counts (n)	Percentage
Age in years (n=193)	19-24	48	25
	25-49	115	60
	50-64	30	15
Gender (n=193)	Woman	125	65
	Man	50	26
	Transgender/Trans	6	3
	Another gender	2	1
	Prefer not to answer	10	5
Ethnic Background (n=193)	Black, African, Caribbean	5	3
	East Asian	7	4
	Latin American	9	5
	Indigenous	4	2
	Native American	7	4
	Middle Eastern	5	3
	South Asian	0	0
	White/European	141	73
	Other/Multiple	12	6
	Prefer not to answer	2	1
Education (n=192)	Did not finish high school	2	1
	High School	20	10
	Some post-secondary	29	15
	Post-secondary	29	15
	Undergraduate Degree	89	46
	Postgraduate degree or professional degree	23	12
Employment Status (n=191)	Employed full-time	81	42
	Employed part-time	28	15
	Self-employed	32	17
	Student	16	8
	Stay-at-home parent	8	4
	Not employed	15	7
	Disability leave	5	3
	Retired	5	3
	Other	2	1
Living Area (n=190)	Urban	105	55
	Suburban	69	36
	Rural or remote area	16	8

Table 2 shows treatment information for the respondents. Most of them were currently taking antidepressants (n=94, 49%) or had taken them in the past (n=92, 48%). Of those currently taking antidepressants (n=94), the majority had taken them for over 6 months (n=73, 78%). In regard to satisfaction with the antidepressant, 62 (66%) respondents were “very satisfied” or “slightly satisfied,” while only 10 (11%) respondents were “slightly unsatisfied” or “very unsatisfied. The majority of participants also reported that they were knowledgeable of depression treatments in general, with 167 (87%) responding “very knowledgeable” or “moderately knowledgeable.” Significantly more of the respondents had heard of light therapy (n=183 [95%]) for depression compared to negative ion therapy (n=121 [63%]; χ2=59.7, df=1, p<0.0001). Also, significantly more participants had used light therapy (n = 56 [29%]) than had used negative ion therapy (n = 32 [17%]; χ2 = 8.3, df = 1, p = 0.004).

**Table 2:** Treatment information

Category	Responses	Count (n)	Percentage
Taking Antidepressants (n=193)	Taking currently	94	49
	Taken in the past	92	48
	Never taken	7	4
Duration of Current Antidepressant Use (n=94)	Less than 6 months	21	22
	6-24 months	28	30
	More than 24 months	45	48
Satisfaction with Current Antidepressant (n=94)	Very satisfied	22	23
	Slightly satisfied	40	43
	Neither satisfied nor dissatisfied	21	22
	Slightly unsatisfied	8	9
	Very unsatisfied	2	2
Knowledge about depression treatment (n=193)	Very knowledgeable	56	29
	Moderately knowledgeable	111	58
	Somewhat knowledgeable	25	13
	Not at all knowledgeable	0	0

**Figure 1:**
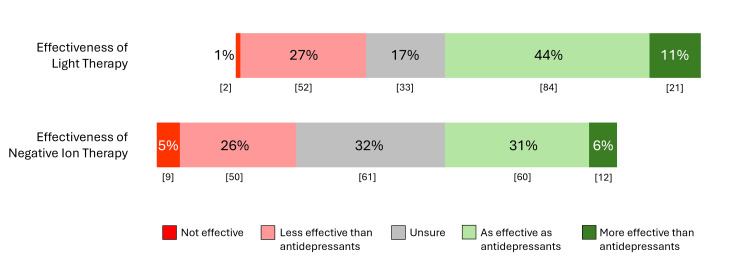
Responses on perceived effectiveness of negative ion therapy and light therapy (n = 192) Numbers in brackets indicate counts.

Figure 1 displays the percentage responses for perceived effectiveness of light therapy and negative ion therapy (Appendix, Q18 and Q20). The number of respondents who regarded light therapy as “more effective” or “as effective” as antidepressants was significantly higher than for negative ion therapy (105 [54%] vs. 72 [37%], respectively; χ2=11.41, df=1, p<0.001). There were also significantly fewer respondents who were “Unsure” of the effectiveness of light therapy than negative ion therapy (33 [17%] vs. 61 [32%], respectively; χ2=11.04, df=1, p<0.001).

**Figure 2:**
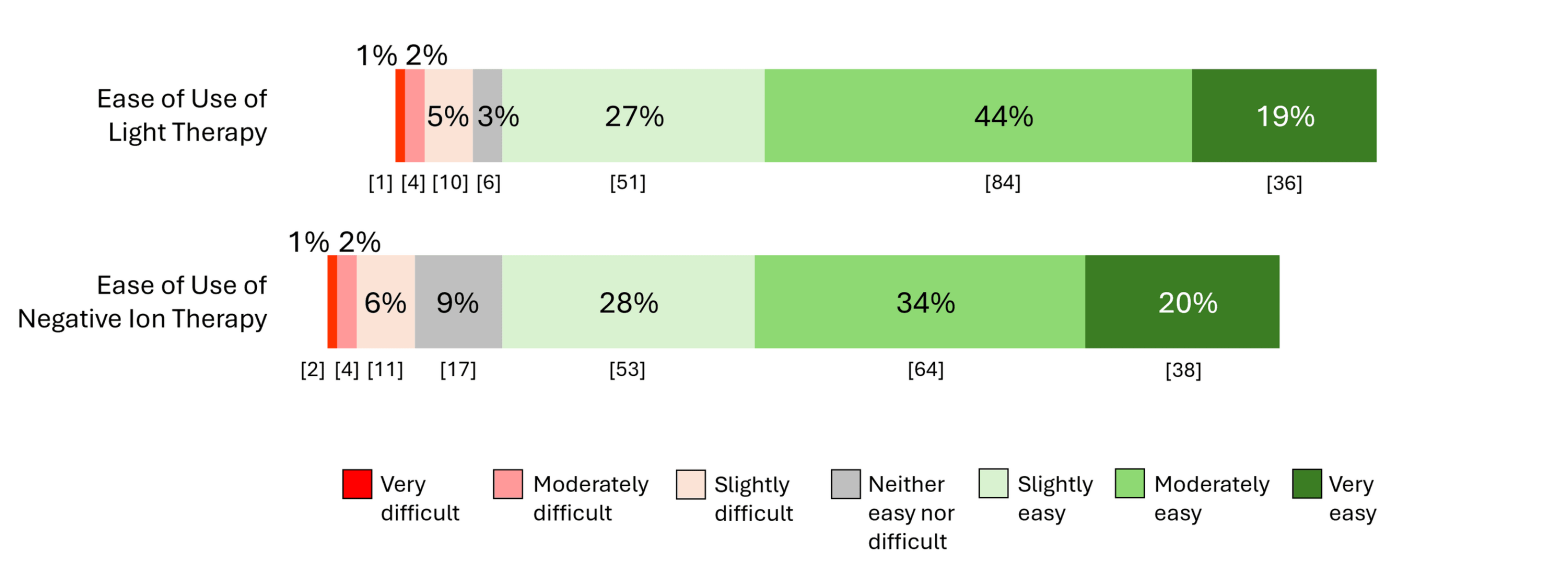
Responses about perceived ease of use of light therapy (n = 192) and negative ion therapy (n = 188) Numbers in brackets indicate counts

Figure 2 displays the responses for perceived ease of use of the two therapies for maintenance treatment (Appendix, Q22 and Q23). There was no significant difference in whether treatments were regarded as easy to use, with 171 (89%) of participants regarding light therapy as “slightly” to “very easy” to use compared to 155 (82%) for negative ion therapy (χ2=3.83, df=1, p >0.05).

Regarding the main reasons for wanting to use and reasons for not wanting to use light therapy or negative ion therapy for maintenance treatment, respondents were offered the opportunity to select as many responses as they wanted (Table 3). The top three individual reasons for wanting to use light or negative ion therapy instead of antidepressants were “it has fewer side effects,” “it is a non-medication treatment,” and “I don’t like taking medications.” The top three individual reasons for not wanting to use light therapy or negative ion therapy were “I’m worried it won’t work,” “I’m worried about withdrawal effects from stopping antidepressants,” and “I don’t have time to do the therapy.”

**Table 3:** Attitudes about treatment * Multiple responses allowed.

Category	Responses	Count (n)	Percentage
Wanting to use light therapy instead of medications*	It is a non-medication treatment	72	37
	It has fewer side effects	92	47
	I don’t like taking medications	61	32
	I have side effects from medication	50	26
	It seems like a natural treatment	46	24
	Other	12	6
Wanting to use negative ion therapy instead of medications*	It is a non-medication treatment	67	35
	It has fewer side effects	71	37
	I don’t like taking medications	71	37
	I have side effects from medication	61	32
	It seems like a natural treatment	32	17
	Other	7	4
What would stop you from using light therapy instead of medications?*	I’m worried it won’t work	62	32
	I don’t like the side effects	32	17
	I don’t have time to do light therapy	51	26
	I can’t afford to buy a light device	30	16
	I’m worried about withdrawal effects from stopping the antidepressants	48	25
	Other	10	5
What would stop you from using negative ion therapy instead of medications?*	I’m worried it won’t work	73	38
	I don’t like the side effects	33	17
	I don’t have time to do negative ion therapy	51	26
	I can’t afford to buy a negative ion device	31	16
	I’m worried about withdrawal effects from stopping the antidepressants	54	28
	Other	10	5

**Figure 3:**
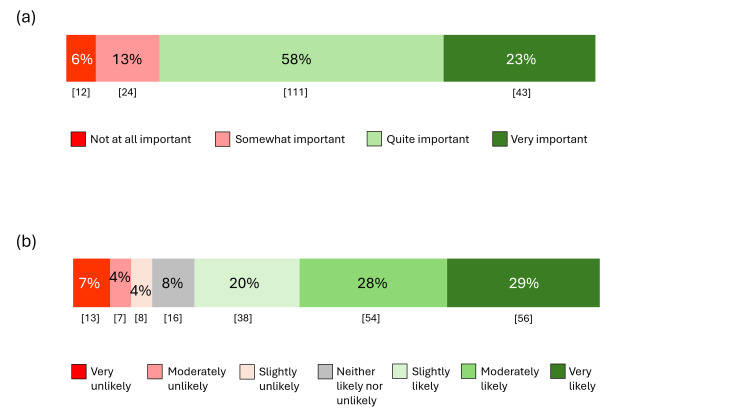
(a) Importance of finding substitute non-medication therapies for maintenance treatment (n=190). (b) Likelihood to volunteer for the randomized study (n=192). Numbers in brackets indicate counts.

Figure 3(a) displays the responses about the importance of finding substitute non-medication treatments for maintenance treatment (Appendix, Q21). Generally, support for the need for non-medication therapies was strong, with 154 (81%) participants identifying it as either “very important” or “quite important”; only 12 (6%) viewed it as “not at all important.” Figure 3(b) displays the responses about an RCT for light and negative ion therapies for maintenance treatment (Appendix, Table 4, Vignette 6, and Q29). A total of 148 (77%) respondents endorsed that they would be “very likely,” “moderately likely,” or “slightly likely” to volunteer for the study.

## Discussion

Patient engagement in research shows evidence for a positive impact, even at early stages of formulating research questions [23,26]. Our online survey of participants who self-reported a diagnosis of depression examined their opinions about light therapy and negative ion therapy and the importance of a trial of maintenance treatment for MDD. The demographic profile of participants (middle-aged, female, White/European ethnicity, university educated) mirrors that of most RCTs for depression treatments. This sample of participants was generally familiar with both treatments, although a significantly higher percentage of participants had heard of light therapy than negative ion therapy (95% vs. 63%, p<0.0001) and had used light therapy (29% vs. 17%, p<0.004). Similarly, a significantly higher percentage of the sample believed that the effectiveness of light therapy was similar to or better than antidepressants compared to negative ion therapy (55% vs. 38%, p<0.001), with more “unsure” about the effectiveness of negative ion therapy (32% vs. 17%, p<0.001). These differences may be expected since light therapy has been widely reported in the media as an effective treatment for SAD and is endorsed by clinical guidelines for depression [2,27], whereas negative ion therapy is still largely limited to research studies.

Both treatments were regarded by respondents as easy to use, with no significant differences between the two. The reasons for wanting to use the treatments were similar across the two therapies, suggesting that they represent general attitudes about non-medication therapies versus medications rather than specifically for one therapy over the other. Having fewer side effects was the top reason for wanting to use both therapies. Reasons for not wanting to use light therapy and negative ion therapy were again similar for both, including apprehension about their effectiveness and about withdrawal effects from stopping antidepressants. Few respondents were concerned about the cost of the devices (16%) or about withdrawal effects when using the light device (25%) or the negative ion device (28%).

Finally, the sample felt strongly about the need for finding alternative non-medication treatments for relapse prevention of depression, with over three-quarters of the sample (81%) finding this an important question to study. A similar percentage of participants (77%) was likely to volunteer for an RCT for maintenance treatment using one of these therapies.

This study is the first to survey people with a diagnosis of depression about their self-reported knowledge, attitudes, and opinions of light and ion therapy as a treatment for depression. The study had limitations, including a non-representative convenience sample recruited by email and social media, which limited the participants to those with internet access. The survey used was researcher-generated, and the questions have not been validated. In addition, the diagnosis of depression was based on self-report, and it is unclear whether the treatments previously used were prescribed or supervised by health care professionals. Finally, as with most online survey studies, we were unable to assess the participants’ level of knowledge about the treatments, their level of engagement with the study vignettes and questions, or their continued focus throughout the process. Nonetheless, given these positive patient survey results, we are now conducting a multicenter RCT (ClinicalTrials, NCT05423275) to address the question of whether these non-medication therapies can substitute for antidepressants as maintenance treatments for depression.

## Conclusions

In conclusion, this online survey showed that people with depression had good familiarity with light therapy and negative ion therapy. Participants expressed positive attitudes towards their potential for maintenance treatment of depression, especially their ease of use. There was endorsement of the importance of finding alternative non-medication treatments to substitute for antidepressants for maintenance treatment, and the majority would likely participate in a randomized study. The findings show that, from a patient perspective, it is important and feasible to investigate the efficacy and safety of light and ion treatments for relapse prevention in patients with MDD. Future research should include the views of patients in the design of clinical trials for these new treatments.

## Appendices

Vignettes

**Table 4:** Selected vignettes, questions and responses from the online survey questionnaire (created by: Raymond W. Lam, Aidan Scott, André Do, Erin E. Michalak, Jill K. Murphy, and Vanessa K. Evans)

Vignette	Question	Responses
Vignette 1: Light therapy is a treatment for depression that uses daily exposure to bright light from a light box device used at home. Light therapy usually has fewer side effects than antidepressant medications.	Q17. Have you heard of light therapy for depression?	(a) Yes, and I have used light therapy (b) Yes (c) No
			Q18. In your opinion, how effective is light therapy for depression treatment?	(a) More effective than antidepressants (b) As effective as antidepressants (c) Less effective than antidepressants (d) Not effective (e) Unsure
	Vignette 2: Negative ion therapy is a treatment for depression that uses daily exposure to negative ions from a negative ion device used at home. Negative ion therapy usually has fewer side effects than antidepressant medications.	Q19. Have you heard of negative ion therapy for depression?			(a) Yes, and I have used negative ion therapy (b) Yes (c) No
			Q20. In your opinion, how effective is negative ion therapy for depression treatment?	(a) More effective than antidepressants (b) As effective as antidepressants (c) Less effective than antidepressants (d) Not effective (e) Unsure	
Vignette 3: Terry is diagnosed with major depressive disorder (clinical depression) by the doctor and is treated with an antidepressant medication. After taking the pills for about three months, Terry is feeling much better and no longer has depressive symptoms. Terry's doctor recommends continuing the antidepressant for another 6-12 months. This “maintenance treatment” will help prevent the depression from returning.						Q21. In your opinion, how important is it to substitute an evidence-based non-medication treatment for antidepressants for maintenance treatment?	(a) Very important (b) Quite important (c) Somewhat important (d) Not at all important
Vignette 4: Instead of taking medications for maintenance treatment, Terry uses light therapy. Terry buys a light box from the pharmacy for about $100 and uses it at home as prescribed. Terry sits in front of the light box for 30 minutes in the morning soon after waking up. Terry can read or eat breakfast while using the light box, but must be awake. Terry uses light therapy every weekday, but not on weekends.		Q22. How easy would it be for you to use light therapy as described?			(a) Very easy (b) Moderately easy (c) Slightly easy (d) Neither easy nor difficult (e) Slightly difficult (f) Moderately difficult (g) Very difficult
Vignette 5: Instead of taking medications for maintenance treatment, Terry uses negative ion therapy. Terry buys a negative ion device from the pharmacy for about $100 and uses it at home as prescribed. Terry sits in front of the negative ion device for 30 minutes in the morning soon after waking up. Terry can read or eat breakfast while using the negative ion device, but must be awake. Terry uses negative ion therapy every weekday, but not on weekends.		Q23. How easy would it be for you to use negative ion therapy as described?			(a) Very easy (b) Moderately easy (c) Slightly easy (d) Neither easy nor difficult (e) Slightly difficult (f) Moderately difficult (g) Very difficult
						Vignette 6: You have been diagnosed with major depressive disorder (clinical depression) by your doctor and have been treated with an antidepressant medication. After taking the pills for about three months, you feel much better and you no longer have depressive symptoms. Your doctor tells you that you should have maintenance treatment for 6-12 months to help prevent the depression from returning. You are now asked to volunteer for a research study to find out whether light therapy or negative ion therapy is effective as a substitute for antidepressants for maintenance treatment. In this study, you will slowly stop taking the antidepressant medication and be randomly assigned (like the flip of a coin) to treatment with light therapy or negative ion therapy for up to 12 months. You will take home the light device or negative ion device to use as described in the previous questions. However, half of the devices have been deactivated so that they are not effective. Therefore, you have a 1 in 2 chance of using an ineffective maintenance treatment. Vignette 6: You will complete questionnaires online (on a computer or mobile device) once a week (which takes about 15 minutes) and will come to the research clinic for a visit once a month (which takes about 1 hour). These questionnaires and visits will check whether your depression symptoms are returning. If the depression symptoms return, you will stop the study and be treated with usual medical care.	Q29. How likely would you be to volunteer for this study?	(a) Very likely (b) Moderately likely (c) Slightly likely (d) Neither likely nor unlikely (e) Slightly unlikely (f) Moderately unlikely (g) Very unlikely
